# Effectiveness and Safety of the Ozaki Procedure for Aortic Valve Disease in Pediatric Patients: A Systematic Review and Meta-Analysis

**DOI:** 10.7759/cureus.45269

**Published:** 2023-09-14

**Authors:** Vikram Halder, Amit Mishra, Soumitra Ghosh, Harkant Singh, Parag Barwad, Shyam K Thingnam, Aduri Raja S Dutta, Maruti Harunal

**Affiliations:** 1 Department of Cardiothoracic Surgery, U. N. Mehta Institute of Cardiology & Research Centre, Gujarat, IND; 2 Department of Cardiothoracic Surgery, U. N. Mehta Institute of Cardiology & Research Centre, Ahmedabad, IND; 3 Department of Cardiology, Postgraduate Institute of Medical Education and Research, Chandigarh, IND; 4 Department of Cardiothoracic Surgery, Postgraduate Institute of Medical Education and Research, Chandigarh, IND; 5 Department of Cardiothoracic Surgery/Congenital Heart Disease, U. N. Mehta Institute of Cardiology & Research Centre, Ahmedabad, IND

**Keywords:** single valve disease, ross procedure, ozaki procedure, children, aortic valve disease

## Abstract

The surgical treatment options for pediatric aortic valve disease are limited and have debatable long-term durability. In the current situation, the Ross procedure is considered in children for aortic valve disease(s). It is a complex surgical procedure with the risk of neo-aortic dilatation, converting a single valve disease into double valve disease, and associated with future re-interventions. Conversely, the Ozaki procedure has shown promising results in adults. Thus, the present study aimed to provide comparative evidence on the effectiveness and safety of the Ozaki versus Ross procedure for pediatric patients by performing a meta-analytic comparison of reporting outcomes. A total of 15 relevant articles were downloaded and among them, seven articles (one prospective study, five retrospective studies, and one case series) were used in the analysis. Primary outcomes such as physiological laminar flow pattern and hemodynamic parameters, and secondary outcomes such as hospital stays, adverse effects, mortality, and numbers of re-intervention(s) were measured in the meta-analysis. There were no significant differences in the age of patients between children who underwent the Ozaki procedure and those who underwent the Ross procedure at the time of surgeries. The Ozaki procedure is a good solution to an aortic problem(s) similar to the Ross procedure. Unlike the Ross procedure, the Ozaki procedure has restored a physiological laminar flow pattern in the short-term follow-up without the bi-valvular disease. Mean hospital stays (*p* = 0.048), mean follow-up (*p* = 0.02), adverse effects (*p *= 0.02), death, and numbers of re-intervention(s) of children who underwent the Ozaki procedure were fewer than those who underwent the Ross procedure. The time required for re-intervention(s) is higher for children who underwent the Ozaki procedure than those who underwent the Ross procedure. None of the procedures, including the Ozaki procedure for aortic valve disease(s), has significant effects on hemodynamic parameters and the frequent death rate of children after surgeries. Based on our analysis, we may suggest the Ozaki procedure for aortic valve disease surgery in children.

## Introduction and background

The Ozaki procedure is aortic valve disease surgery, where custom-tailored neo-aortic valve cusps are prepared from glutaraldehyde-treated autologous pericardium and sutured at the aortic valve [1]. The Ozaki procedure received Food and Drug Administration approval in the USA in 2014 [2] and has the benefits of excellent postoperative hemodynamic parameters, preservation of natural expansion of the aortic root in systole, provision of the maximum effective aortic valve orifice area, and no need for anticoagulants treatment [1]. In adults, for aortic valve disease, the replacement of the aortic valve is the gold standard whereas the prosthetic valve has issues of hemodynamic parameters [3]. The surgical treatment options for pediatric aortic valve disease are limited [2]. In children, the biological prosthetic has a rapid rate of deterioration [4, 5]. Therefore, balloon valvuloplasty, surgical valve repair, and the Ross procedure (aortic autograft) are generally preferred in children [1,6]. Also, there is a risk of prosthetic valve failure after aortic valve disease surgery in children [7]. In the current situation, the Ross procedure is considered in children for aortic valve disease(s) surgery but it is a complex surgical procedure [6]. There is the risk of autograft dilation within the first two decades in the patients who underwent the Ross procedure [8]. Thus, the present study aimed to provide comparative evidence on the effectiveness and safety of the Ozaki versus Ross procedure for pediatric patients by performing a meta-analytic comparison of reporting outcomes.

## Review

Materials and methods

Search Strategy

Research and review articles, case reports, and meta-analyses published in 2010 or after that on children (<18 years) were collected using the mixed terms 'Ozaki and Ross procedures', ‘Ozaki procedure’, ‘pediatric Ozaki procedure’, 'Ross procedure' and “Autologous pericardial reconstruction of aortic valve” of in PubMed, Google Scholar, Science Direct, and Cochrane Library. Research and review articles, case reports, and meta-analyses before 2010, those regarding adult patients, those on the Ross procedure only, and those in the other than English language were excluded from the analysis.

Statistical Analyses

InStat 3.01 (GraphPad Software, San Diego, CA, USA) was used for statistical analyses purpose. Continuous data are presented as mean and constant data are presented as frequency (percentage). Student t-test or Mann-Whitney test and Fisher exact test were performed for statistical analyses. A p-value less than 0.05 was considered significant.

Results

**Figure 1:**
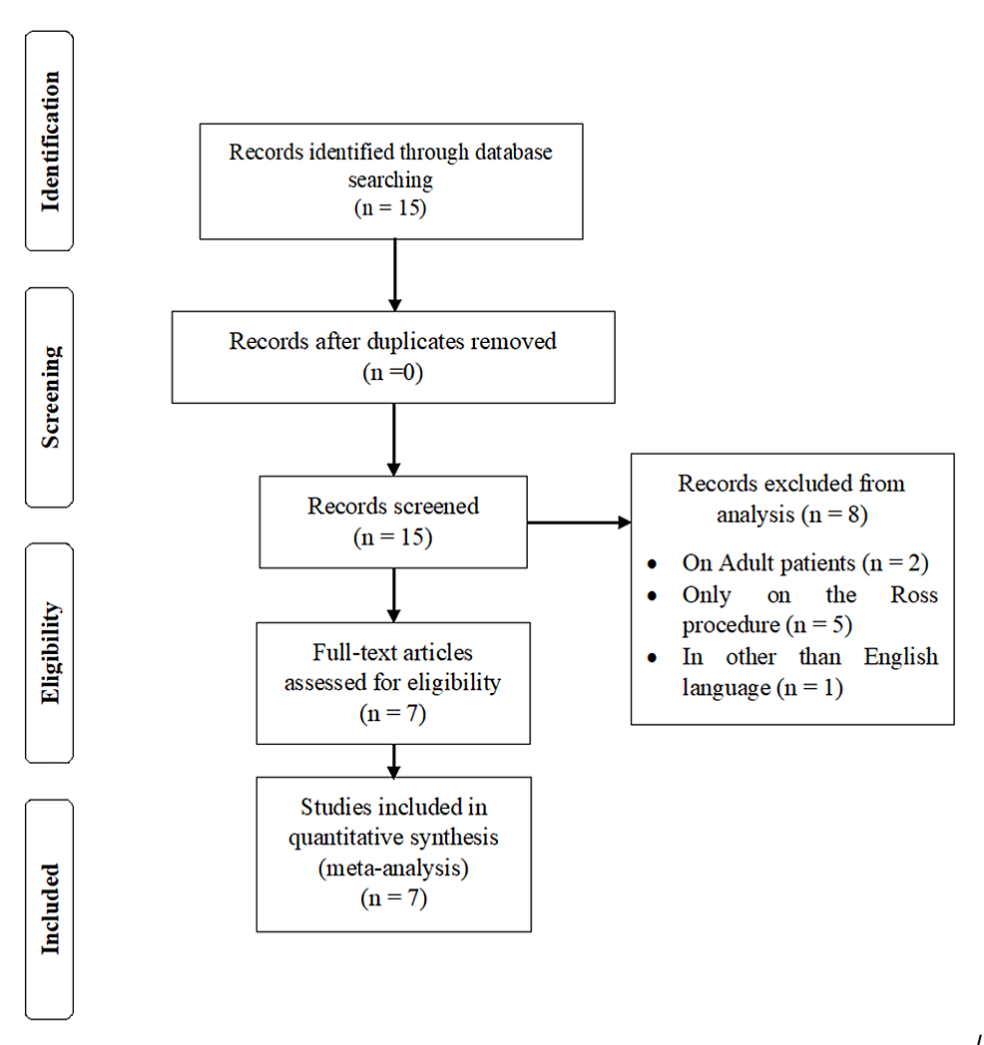
PRISMA flow chart of the study. PRISMA: Preferred Reporting Items for Systematic Reviews and Meta-Analyses

A total of 15 relevant articles were downloaded and among them, seven articles were used in the analysis [1-15] and eight articles were excluded. The Preferred Reporting Items for Systematic Reviews and Meta-Analyses (PRISMA) flow chart of meta-analyses is presented in Figure 1.

One prospective study, five retrospective studies, and one case series were included in the analyses. All included articles have both male and female patients. Hemodynamic parameters of patients before surgeries were stable. There were no significant differences in the age of patients between children who underwent the Ozaki procedure and those who underwent the Ross procedure at the time of surgeries. The characteristics of patients of studies included in the meta-analysis are reported in Table 1.

**Table 1:** Characteristics of patients of studies included in the meta-analysis M: Male, F: Female, N/A: Not applicable, N/V: Not available.

Authors, year	Country	Study period	Study design	Numbers of patients	Mean Age of patients	Sex (M/F)	Indications	Age at the time of surgeries		
								The Ozaki procedure	The Ross procedure	p-value
Chivers et al., 2019 [1]	United Kingdom	January and July 2016	Case series	5	17.60(range: 11–29) years	4/1	Aortic valve disease (3); aortic regurgitation alone (2)	17.60±6.99 years	17±5.66 years	0.919
Polito et al., 2020 [9]	Italy	January 2015 to May 2020	Retrospective	38	12.40(8.8–15.8) years	-	Aortic valve stenosis (20); aortic valve incompetence (18)	13.9 (9.8–16.2) years	11.1 (6.6–14)	0.14
Baird et al., 2021 [10]	USA	August 2015 to February 2019	Retrospective	57	12.43 years	21/36	Aortic regurgitation (24); aortic stenosis (6); both (27)	12.43 years	N/V	N/A
Secinaro et al., 2021 [11]	Italy	-	Prospective	20	10.7(range 3.9–16.5) years	15/5	Aortic valve defect	10.6 ± 4.2 years	11.4 ± 3.1 years	0.64
Marathe et al., 2020 [12]	USA	2015 to 2019	Retrospective	51	7.90 years(7 months–25 years)	36/15	Aortic regurgitation (23); aortic stenosis (22); both (6)	7.90 years(7 months–25 years)	N/V	N/A
Wiggins et al., 2020 [13]	United Kingdom	2015 to 2019	Retrospective	58	18.8 years(10.6–16.8) years	N/V	Aortic stenosis (14); mixed pathology (21); Aortic regurgitation (23)	N/V	N/V	N/A
Polito et al., 2021 [14]	Italian	July 2016 to May 2020	Retrospective	22	13.9 years (9.8–16.2 years)	N/V	Aortic valve stenosis (10); Aortic valve incompetence (12)	N/V	N/V	N/A

Primary Outcome

The Ozaki procedure is a good solution to an aortic problem similar to the Ross procedure. However, unlike the Ross procedure, the Ozaki procedure has restored a physiological laminar flow pattern in the short-term follow-up without the bi-valvular disease. None of the procedures has significant effects on hemodynamic parameters. The primary parameters of patients in the included studies are reported in Table 2. 

**Table 2:** Functional parameters of patients of the included studies

Authors, year	Follow-up time	Functional parameters		Conclusions
		For Ozaki procedure	For other than the Ozaki procedure	
Chivers et al., 2019 [1]	29.6 (range: 22–36) months	No regurgitation from the neo-aortic valve	Good cardiac function (the Ross procedure)	The Ozaki procedure should be approached with caution
Polito et al., 2020 [9]	18.2 (range: 5–32) months	Effective treatment of aortic problem	Effective treatment of aortic problem (the Ross procedure)	The Ozaki technique is a more suitable strategy
Baird et al., 2021 [10]	8.1 (range: 2.96–17.76) months	Acceptable short-term results	-	An effective technique for valve reconstruction in pediatric patients
Secinaro et al., 2021 [11]	4 (1–10) months	Restores a physiological laminar flow pattern in the short-term follow-up without bi-valvular disease	Restores a physiological laminar flow pattern in the short-term follow-up with bi-valvular disease(the Ross procedure)	An effective technique to restore physiological tri-leaflet fluid-dynamic conditions
Marathe et al., 2020 [12]	11.9 (1 month–2.6 year)	Effective treatment of aortic problem	-	The Ozaki procedure is a good option for standard valve replacement
Wiggins et al., 2020 [13]	4 years	After surgery, the gradient across the aortic valve is decreased and remained stable with delay time of re-operation.	After surgery the gradient across the aortic valve is decreased and remained stable but earlier chances of reoperation (Single leaflet reconstruction)	Aortic leaflet reconstruction techniques can be applied in children and young adults
Polito et al., 2021 [14]	11.3 (4.7–21) months	The Ozaki procedure is a safe technique that allows satisfactory resolution of aortic valve diseases in children	A safe technique(Rankin approach + modified Yamaguchi approach)	The Ozaki procedure is safe and effective in pediatric patients with aortic valve disease

Secondary Outcome

Mean hospital stays (p = 0.048), mean follow-up (p = 0.02), and adverse effects (p = 0.02) of children who underwent the Ozaki procedure were fewer than those who underwent the Ross procedure. The deaths in follow-up are fewer for the Ozaki procedures than that in any of the surgical procedures but data are not statistically significant between the Ozaki procedure and any of the surgical procedures. Similarly, fewer numbers of re-intervention(s) and a higher time for re-intervention(s) are required for the Ozaki procedure as compared to those of any of the surgical procedures. However, values for the number of re-intervention(s) and time for re-intervention(s) are not statistically significant between the Ozaki procedure and any of the surgical procedures. The secondary parameters of patients of the included studies are reported in Table 3.

**Table 3:** Secondary outcome of patients the included studies N/A: Not applicable, N/V: Not available. Continuous data are presented as mean (range) and constant data are presented as frequency (percentage). Student t-test or Mann-Whitney test and Fisher exact test were performed for statistical analyses. A p-value less than 0.05 was considered significant. *: Significant value.

Parameters	Procedures and comparisons	Authors, year						
		Chivers et al., 2019 [1]	Polito et al., 2020 [9]	Baird et al., 2021 [10]	Secinaro et al., 2021 [11]	Marathe et al., 2020 [12]	Wiggins et al., 2020 [13]	Polito et al., 2021 [14]
Children distributions	The Ozaki procedure	5	12	57	10	51	40	18
	The Ross procedure	2	16	0	10	0	18 (Single leaflet reconstruction)	4 (Rankin approach + modified Yamaguchi approach)
Mean hospital stays (days)	The Ozaki procedure	8.2	8(7–11)	6.4(5.25–7.36)	N/V	7.2(3.5–293)	5(4–7)	8 (7–11)
	The Ross procedure	N/V	8(7–14.5)	N/V	N/V	N/V		
	p-value	N/A	0.048^*^	N/A	N/A	N/A	N/A	N/A
Mean follow-up time	The Ozaki procedure	29.6 months	11.3(4.7–21) months	8.1 months	4(1–10) months	11.9(1 month–2.6 year)	N/V	11.3 (4.7–21) months
	The Ross procedure	N/V	38.9 (13.8–52.8) months	N/V	4(1–10) months	N/V	N/V	
	p-value	N/A	0.02^*^	N/A	N/A	N/A	N/A	N/A
Adverse effects	The Ozaki procedure	Complications related to the valve (2 out of 5)	Required aortic valve replacement (3 out of 22)	Mild or less regurgitation and peak aortic gradient	Transvalvular maximum velocity: 220±73 cm/s	Required surgical re-intervention	Re-intervention and time 4(10) children (11.6±12.45; 9.1–27.8 months)	2 (11) valve replacement
	The Ross procedure	Transient ischemic attack (1 out of 2)	Required aortic valve replacement (1 out of 16)	N/V	Transvalvular maximum velocity: 130±33 cm/s	N/V	Re-intervention and time 2(11) children (16.88±8.60; 2.8–20.4 months) (Single leaflet reconstruction) (Single leaflet reconstruction)	0(0) (Rankin approach + modified Yamaguchi approach)
	p-value	0.999	0.625	N/A	0.02^*^	N/A	0..999 for numbers of re-intervention (0.8 for re-intervention time)	0.999
Death	The Ozaki procedure	0(0)	0(0)	2(4)	0(0)	0(0)	1(3)	N/V
	The Ross procedure	0(0)	1(16)	N/V	0(0)	N/V	0(0) (Single leaflet reconstruction)	N/V
	p-value	N/A	0.421	N/A	N/A	N/A	0.999	N/A

Discussion

Unlike adults, children have different postoperative outcome measures [14]. A higher rate of mortality and postoperative complications are reported for different aortic valve disease(s) surgical procedures [14]. Therefore, the Ross procedure is the gold standard for aortic valve disease surgery but the Ozaki procedure has also reported effective short-term results without the bi-valvular disease [11]. The dilatation of neoaortic root after the Ross procedure is responsible for the bi-valvular disease. This is not found in the Ozaki procedure [6]. Also, the Ross procedure has mortality and postoperative complications like the other aortic valve disease(s) surgical procedures [14]. Ross procedure is not feasible in patients with post arterial switch and truncus arteriosus but Baird et al described Ozaki reconstruction in truncus patients [10]. However, the Ozaki procedure is reported with good postoperative results and less mortality [1,9,11] and it has adequate intraoperative hemodynamic results [10]. The Ozaki procedure is a good option for aortic valve disease(s). There are different options for patches like treated autologous pericardium, Photofix (CryoLife, Kennesaw, USA)**, **CardioCel (Admedus Ltd., Toowong​​​​​​​, Australia), and Matrix (Auto Tissue Berlin GmbH, Berlin, Germany). These are helpful in redo cases. As Matrix is thinly sliced equine untreated pericardium, it can be a good option for the paediatric population [13].

Hospital stays [9], adverse effects [1,11], follow-up time [9], morbidity after surgeries [9], and death for children who underwent the Ozaki procedure are less than those who underwent the Ross procedure or any other procedure for aortic valve disease(s). In the Ozaki procedure, there is less postoperative bleeding with respect to the Ross procedure. One of the disadvantages of the Ross procedure is that the single valve disease is converted to double valve disease [6]. Also, the age of children influences aortic root dimensions and aortic regurgitation during the Ross procedure [15]. 

Freedom from reoperation (or moderate and greater aortic regurgitation) is reported for children who underwent the Ozaki procedure [13,14]. A lower reoperation rate is related to fewer adverse effects and mortality for the Ozaki procedure [9]. Unlike the Ross procedure and any other procedure for aortic valve disease(s), the Ozaki procedure has fewer chances of reoperation and mortality. But theoretically, in the Ross procedure, the autograft can grow with age but not possible with autologous pericardium. Long-term data is missing in the Ozaki procedure in children. Blitzer et al described about aortic valve neocuspidization in pulmonary autograft because of aortic regurgitation [16].

Limitations

There are limitations of the study, for example, meta-analysis (indirect evidence) and lack of prospective study (direct evidence). In younger patients, the results of the outcome measures are different than those of adult patients [2], but the long-term results in pediatric patients for the Ozaki procedure are not reported. 

## Conclusions

Based on the analysis, we found that the Ozaki procedure has better functional parameters and postoperative parameters than the Ross procedure and any other procedure for aortic valve disease(s). Therefore, we may suggest the Ozaki procedure as a considerable treatment option for aortic valve disease surgery in children.
